# Accurate protein structure prediction with hydroxyl radical protein footprinting data

**DOI:** 10.1038/s41467-020-20549-7

**Published:** 2021-01-12

**Authors:** Sarah E. Biehn, Steffen Lindert

**Affiliations:** Department of Chemistry and Biochemistry, Ohio State University, Columbus, OH 43210 USA

**Keywords:** Structural biology, Computational models, Biochemistry

## Abstract

Hydroxyl radical protein footprinting (HRPF) in combination with mass spectrometry reveals the relative solvent exposure of labeled residues within a protein, thereby providing insight into protein tertiary structure. HRPF labels nineteen residues with varying degrees of reliability and reactivity. Here, we are presenting a dynamics-driven HRPF-guided algorithm for protein structure prediction. In a benchmark test of our algorithm, usage of the dynamics data in a score term resulted in notable improvement of the root-mean-square deviations of the lowest-scoring ab initio models and improved the funnel-like metric P_near_ for all benchmark proteins. We identified models with accurate atomic detail for three of the four benchmark proteins. This work suggests that HRPF data along with side chain dynamics sampled by a Rosetta mover ensemble can be used to accurately predict protein structure.

## Abstract (web summary)

Mass spectrometry-based covalent labeling techniques such as hydroxyl radical protein footprinting (HRPF) provide information about protein tertiary structures. Here, the authors present a dynamics driven HRPF-guided algorithm for protein structure prediction that is incorporated in the Rosetta software suite and only requires the protein sequence and HRPF data as input and they demonstrate its successful application to four benchmark proteins.

## Introduction

In addition to sequencing and elucidating the mass to charge ratio of proteins, mass spectrometry data can provide insight into protein structure^1–5^. Currently, a number of techniques exist with the potential to determine elements of protein tertiary structure^6^. Hydrogen–deuterium exchange involves the exchange of solvent deuterium atoms with amide hydrogen atoms, and structural changes are easily identified by the addition of one atomic mass unit^7^. Limited proteolysis relies on the partial cleavage of the protein at particular residues, with residues that are more exposed being more likely to be enzymatically cleaved^8^. Chemical crosslinking facilitates the cross-linking of protein functional groups both within protein subunits and within complexes^9^. Covalent labeling involves exposure of a protein to a labeling reagent that will irreversibly modify residues. Residues that are more accessible to solvent are generally more likely to be covalently modified, providing insight into the tertiary protein structure^10,11^.

Covalent labeling can be achieved with a multitude of reagents, including carbenes, diethylpyrocarbonate, and hydroxyl radicals^11–13^. Hydroxyl radicals, a commonly used covalent labeling reagent, are frequently derived from radiolysis or photolysis of hydrogen peroxide or water^14,15^. Hydroxyl radical protein footprinting (HRPF) is an attractive labeling method because of its high sensitivity, robustness, and simplicity^16^. Also, the majority of the amino acids can be covalently modified with varying degrees of reactivity and reliability^16,17^.

Despite the promising utility of HRPF, the covalent labeling data lacks detailed structural information sufficient for unambiguous tertiary structure determination^18^. The marriage of HRPF data and computational techniques provides a unique opportunity for more accurate protein structure prediction. The recent innovative work of Xie and colleagues featured molecular dynamics (MD) simulations used cooperatively with HRPF protection factor (PF) data^19^. A strong correlation between experimentally determined PFs and solvent accessible surface area calculated from the MD simulations was observed. Notably, this agreement could be used to accurately distinguish models with backbone root-mean-square deviation (RMSD) greater than 4 Å from models with backbone RMSD less than 3 Å. For the first time, this validated the capability of using HRPF data to identify low RMSD computational models^19^. We have previously shown that the correlation between experimentally determined PFs and residue neighbor count can be exploited as a Rosetta scoring term to improve protein structure prediction^20^. In addition, computational protein structure prediction guided with sparse experimental data has been successfully implemented for a wide range of experimental data^21–27^. Our previous HRPF modeling work, however, relied on static protein structures. Because proteins under physiological conditions are not strictly static objects and sample ensembles of protein conformations, we hypothesized that accounting for protein flexibility could improve the correlation between residue solvent exposure metric and experimental covalent labeling data and hence protein structure prediction.

In this study, we have probed whether the incorporation of protein dynamics can improve previously observed correlations between residue neighbor counts and experimentally derived PFs. We developed a scoring term that uses HRPF data and rewards models using a dynamics-based agreement of their PFs and conical neighbor counts. For the benchmark protein ab initio models, our score term improved model quality and *P*_near_ value upon rescoring with HRPF data versus Rosetta’s score function alone. In addition, Rosetta movers were used to generating ensembles of models of the top ab initio structures. The best scoring model RMSD improved considerably for all four proteins in our benchmark set. We identified models with accurate atomic detail for three out of four benchmark proteins, indicating that factoring dynamics into the prediction played a strong role in our enhanced results.

## Results and discussion

### Initial optimization of the correlation between experimentally derived PF and conical neighbor count

**Fig. 1:**
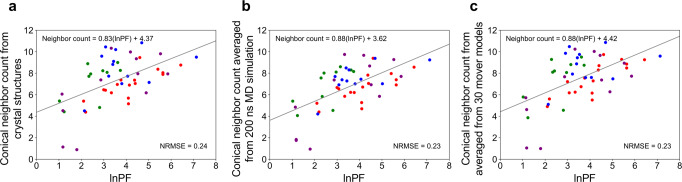
Correlations of the natural logarithm of the protection factor with conical neighbor count using residue types WYFHL. The line of best fit, equation of the line, and the NRMSE value are included in the plot. The labeled residues are color-coded by protein. Myoglobin labeled residues are red, calmodulin green, lysozyme blue, and LMPTP purple. **a** Conical neighbor count was calculated from crystal structures of benchmark proteins. **b** Conical neighbor count was averaged over every frame of a 200 ns MD simulation. **c** Conical neighbor count has averaged over 30 Rosetta mover models per benchmark protein.

Our first goal was to improve the correlation between residue neighbor count and HRPF data. Our benchmark set consisted of four proteins (myoglobin, calmodulin, lysozyme, and low molecular weight protein tyrosine phosphatase (LMPTP)). These proteins had at least 15 labeled residues with residue-resolved PF data available^19,28,29^. To optimize the conical neighbor count calculation, we tested and identified an angle midpoint value of *π*/2 that balanced minimized NRMSE and larger *R*^2^ values (Supplementary Table 1). We further systematically tested residue types grouped by their relative intrinsic reactivities^30^. We found that residue types W, Y, F, H, and L yielded a low NRMSE value (NRMSE = 0.24) for the natural logarithm of the PF (lnPF) and conical neighbor count calculated from crystal structures of benchmark proteins (Fig. 1a and results for other residue types in Supplementary Fig. 1). These five residue types are characterized by high to intermediate relative intrinsic reactivity, defined as 5–20 times the intrinsic reactivity of Pro. This might make them the most useful residues in HRPF, and we speculated that this intrinsic reactivity range played a role in the lower NMRSE value of this combination of residues compared to more extensive residue type sets.

Because we hypothesized that a failure to account for protein dynamics may contribute to a disagreement between lnPF and conical neighbor count, we simulated dynamics with NAMD and a Rosetta mover ensemble^31–33^. Averaging conical neighbor count from a 200 ns simulation over frames extracted every 2, 10, 20, 30, 50, 100, 500, or 1000 ps showed little change in NRMSE values (Supplementary Table 2). Overall, conical neighbor count averaged from the 200 ns simulation for all proteins had a slightly lower NRMSE value (0.23) than that from crystal structures (Fig. 1b). Since sidechain dynamics occur on a 1–10 ns timescale, the 200 ns simulation length should adequately capture sidechain fluctuations that could influence labeling^34^. Because hundreds of nanoseconds MD simulations are computationally expensive and time-consuming, we strove to account for side-chain flexibility at a fraction of the cost via a Rosetta mover ensemble. Our mover set combined a normal mode analysis mover with the *FastRelax* mover to sample various protein conformations based on the crystal structure. We generated 10, 20, 30, 40, 50, 100, 150, and 200 models per benchmark protein and averaged conical neighbor counts overall models, then calculated NRMSE from the correlation with lnPF (Supplementary Table 2). Averaging conical neighbor count from thirty models per benchmark protein generated with movers led to a lower NRMSE (0.23) value correlated with lnPF than with neighbor count from crystal structures (Fig. 1c). A comparison between the conical neighbor counts of labeled WYFHL residues from crystal structures, MD frames, and mover models is shown in Supplementary Fig. 2.

Neighbor counts from MD and the Rosetta mover set had comparable NRMSE values when related to lnPF but at vastly different computational costs. All processors used for computations were model type Intel^®^ Xenon^®^ CPU E5-2650 v4 at 2.20 GHz. Totally, 200 ns MD simulations took about 52 h per protein system using the Ohio Supercomputer Center^35^. The NAMD simulation production runs utilized 28 CPUs with GPU acceleration, requiring about 1456 core hours per protein system. For comparison, if 28 processors were used to generate 30 structures with the Rosetta mover set, about 0.3 h would be required. Compared to the MD simulations, the Rosetta mover ensemble resulted in a 173-fold decrease in both run time and computational cost, allowing for Rosetta mover results to be obtained more quickly than NAMD results. We speculated that the Rosetta mover ensemble can capture some of the relevant side-chain dynamics in less time than the MD simulation. While the changes in NRMSE were not statistically significant, we aimed to pursue structure prediction with the equation relating lnPF and conical neighbor count averaged from mover models as we hypothesized that the implementation of dynamics would lead to downstream improvements in structure prediction.

### Scoring model agreement with HRPF data improved RMSD of the lowest-scoring model and the *P*_near_ value

**Fig. 2:**
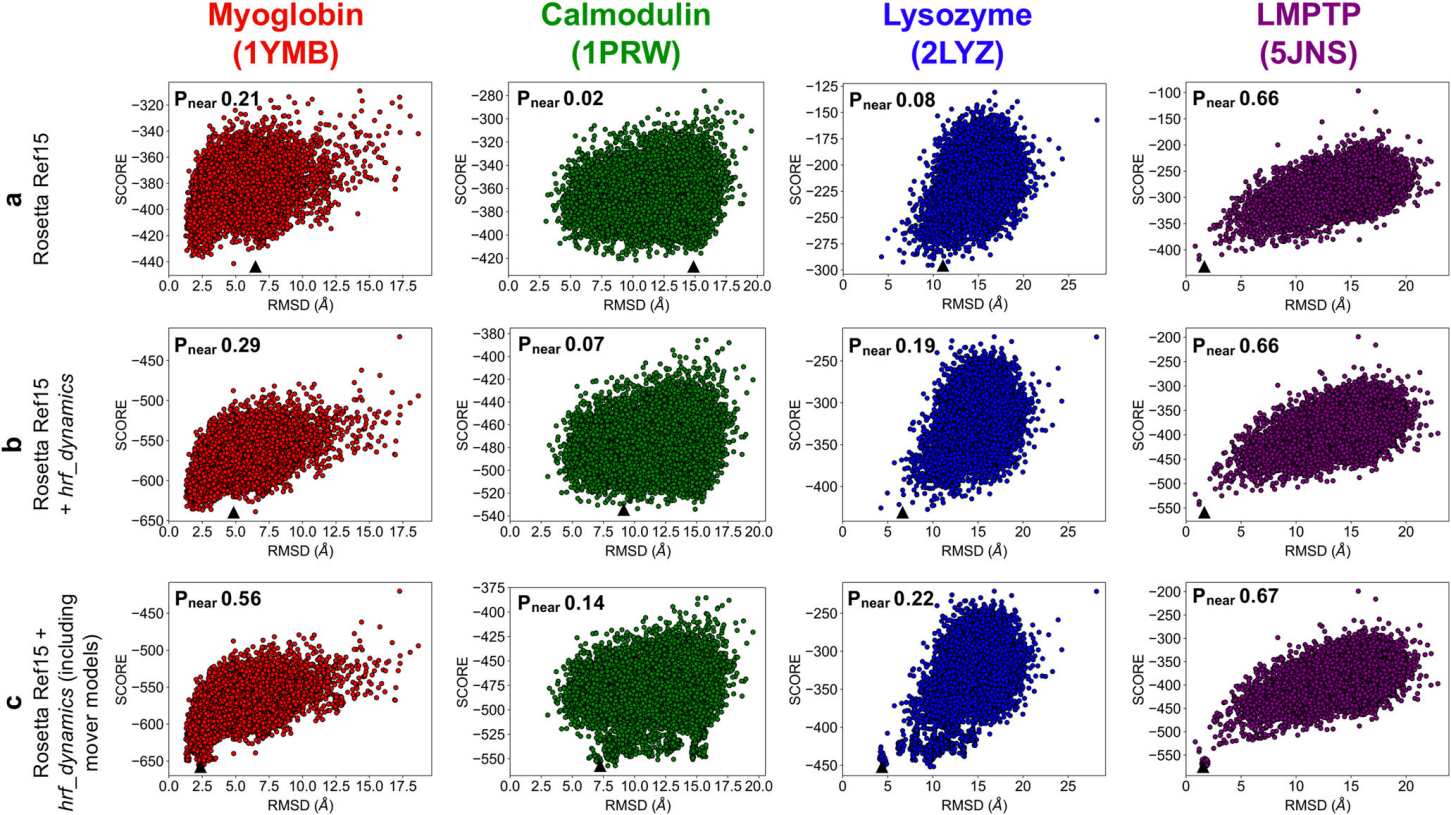
Score versus RMSD to the crystal structure for 20,000 ab initio models generated for each of the four benchmark proteins with the top-scoring model marked by a black triangle. The *P*_near_ value is denoted on each plot. Myoglobin models are shown in red, calmodulin in green, lysozyme in blue, and LMPTP in purple. **a** Rosetta Ref15 score versus RMSD. **b** Rosetta Ref15 + *hrf_dynamics* total score versus RMSD. **c** Rosetta Ref15 + *hrf_dynamics* total score versus RMSD, including the 30 mover models generated per structure for the top 20 scoring models.

**Fig. 3:**
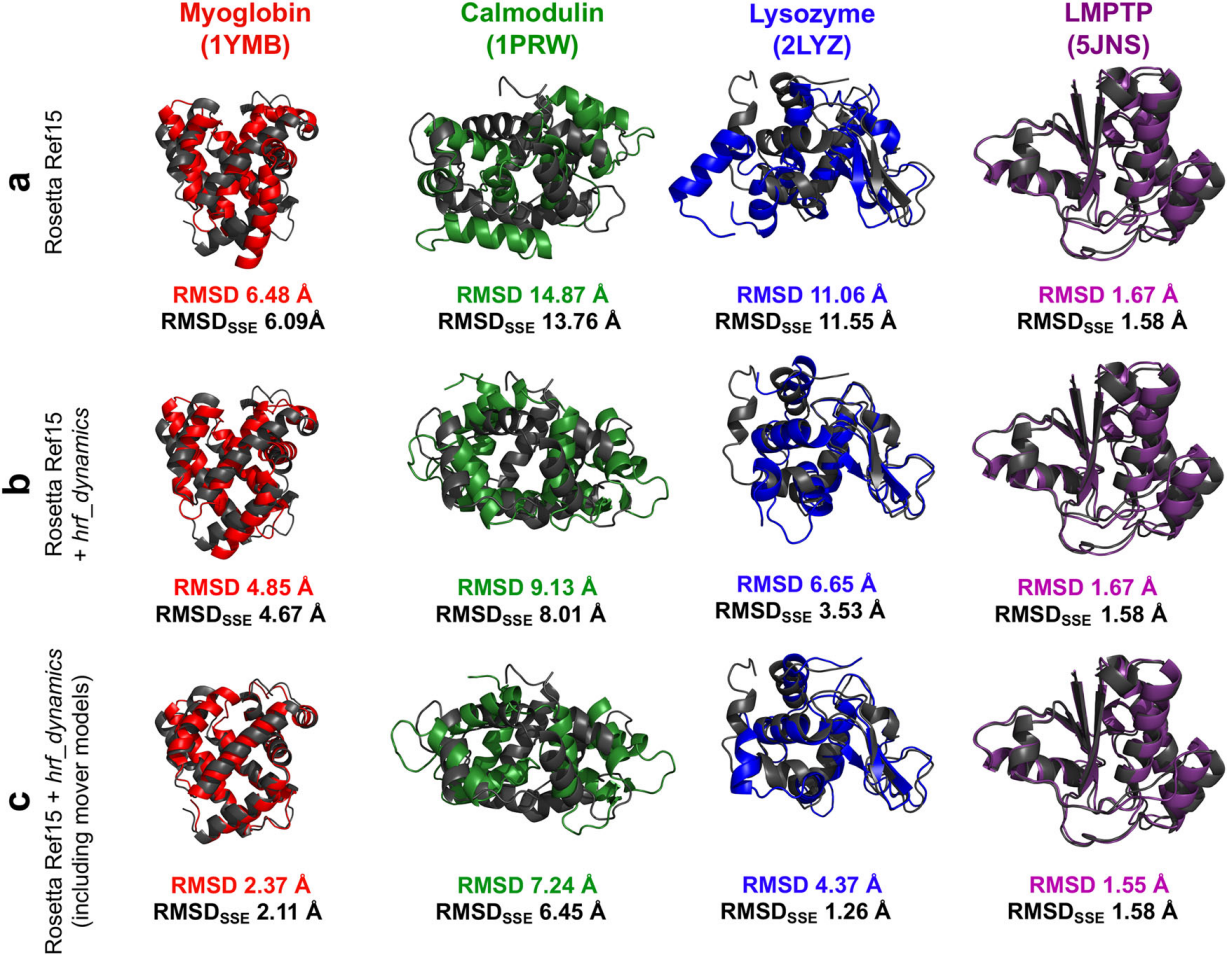
Crystal structures (dark gray) of benchmark proteins aligned with best scoring model predictions (color). RMSD and RMSD over SSEs (RMSD_SSE_) are listed. Structure visualization was accomplished in PyMOL. Myoglobin models are shown in red, calmodulin in green, lysozyme in blue, and LMPTP in purple. **a** Best scoring model based on scoring with Rosetta Ref15. **b** Best scoring model based on scoring with Rosetta Ref15 + *hrf_dynamics*. **c** Best scoring model including mover models scored with Rosetta Ref15 + *hrf_dynamics*.

We used the equation relating lnPF and conical neighbor count averaged from the mover models for implementation of an improved Rosetta HRPF scoring term. Our main goal was to further enhance top-scoring model quality with HRPF data by accounting for dynamics in our prediction equation. While we relied on crystal structures for our initial analysis and determination of our prediction equation for rescoring, we did not rely on crystal structures for any of the actual modeling. We investigated the equation’s prediction capability by comparing observed and predicted neighbor counts for each benchmark protein’s ten top-scoring ab initio models with RMSD within 5 Å of the best RMSD model generated (top-scoring low RMSD models) and ten top-scoring ab initio models with RMSD greater than 10.0 Å (top-scoring high RMSD models). We quantified the difference between observed and predicted neighbor counts by determining how the percentage of labeled residues compared between the low RMSD and high RMSD sets (Supplementary Fig. 3). We selected a deviation value, delta, of 3.5 to be used in the scoring term, as this value captured the upper end of the range of high difference between low and high RMSD models. A larger delta value resulted in more residues being scored, providing a meaningful contribution to the largest possible number of labeled residues. We then proceeded to evaluate the scoring term by examining whether it could enhance structure prediction quality. 20,000 ab initio models were generated for each benchmark protein. The models were then rescored with our score term, *hrf_dynamics*, which was added to the original Rosetta Ref15 score. While we examined weight values from 1 through 20, values greater than or equal to 12.0 were found to consistently maximize improvement for benchmark proteins (Supplementary Fig. 4). Total score versus RMSD to the crystal structure plots are shown in Fig. 2; Rosetta Ref15 scores versus RMSD are shown in Fig. 2a while Rosetta score + *hrf_dynamics* versus RMSD plots are shown in Fig. 2b. As seen in the density scatter plots shown in Supplementary Fig. 5, there was a high density of models at high RMSD values, and lower RMSD models were comparatively rare. Supplementary Fig. 6 includes the *hrf_dynamics* versus RMSD plots. Interestingly, the *hrf_dynamics* score term alone was not necessarily more funnel-like than the Ref15 scoring function and indicative of the trends observed when the scoring term was combined with Rosetta Ref15. However, most individual Rosetta score terms (the components of Ref15) are not funnel-like individually and rely on being combined with other terms to form the Rosetta Ref15 score^36^. In addition, data from HRPF experiments is not comprehensive of protein structure, so combining Rosetta Ref15 + *hrf_dynamics* led to results with the enhanced structural agreement. Figure 3a, b shows the crystal structure aligned with the top-scoring model from Rosetta scoring and rescoring with *hrf_dynamics,* respectively. Compared to scoring with Rosetta Ref15, the addition of *hrf_dynamics* term to the score improved all metrics by which we quantified scoring. The best scoring model RMSD for LMPTP remained at 1.67 Å when the *hrf_dynamics* term was used. As this model already contained accurate atomic detail, it was reassuring that rescoring did not increase the best scoring model RMSD. Rescoring of calmodulin models showed a fair improvement, with the best scoring model RMSD improving from 14.87 to 9.13 Å. Improvement was observed for lysozyme, for which the best scoring model RMSD decreased from 11.06 to 6.65 Å, with the lowest RMSD model generated for lysozyme being 4.24 Å. The best scoring model RMSD for myoglobin improved from 6.48 to 4.85 Å.

In addition to improving the RMSD of the best scoring model, the application of the *hrf_dynamics* score improved the funnel-like quality of the score versus RMSD distribution. We assessed funnel-like quality with *P*_near_; a *P*_near_ value of 1.0 indicates perfect funnel-like quality while a *P*_near_ value of 0 indicates no funnel-like quality^37^. The *P*_near_ values improved for all four benchmark proteins with rescoring, signifying an increase in the funnel-like nature of the score versus RMSD plots. The *P*_near_ for calmodulin, while starting at 0.02, did show improvement by increasing to 0.07 with rescoring. The lysozyme *P*_near_ improved from 0.08 to 0.19 with rescoring. The myoglobin *P*_near_ value increased from 0.21 to 0.29, while the LMPTP *P*_near_ stayed constant at 0.66. We also compared the RMSD of the top-scoring models to the crystal structure for residues involved in secondary structural elements (SSEs). LMPTP RMSD_SSE_ stayed constant at 1.58 Å for the top-scoring model before and after rescoring. Calmodulin RMSD_SSE_ for the top-scoring model improved from 13.76 Å with Rosetta to 8.01 Å after rescoring. The RMSD_SSE_ for myoglobin improved from 6.09 to 4.67 Å. Lysozyme showed a notable improvement, with the RMSD_SSE_ improving from 11.55 Å with Rosetta top model to 3.53 Å with the rescoring top model. This underlined that rescoring with *hrf_dynamics* successfully improved top-scoring model quality.

**Fig. 4:**
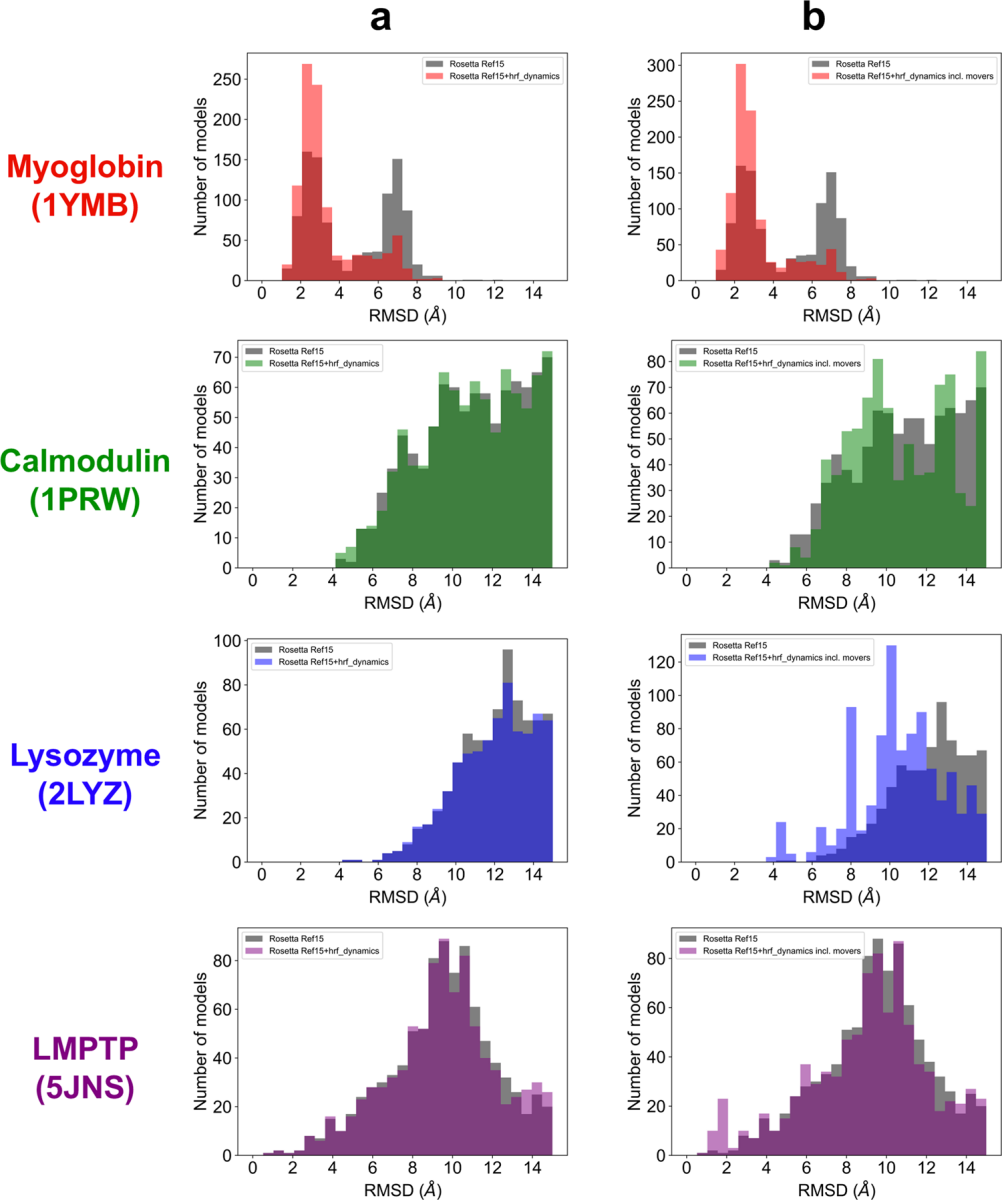
RMSD histograms for each benchmark protein showing the top 1,000 scoring models. RMSD values range from 0.0 to 15.0 Å with bin widths of 0.5 Å. Myoglobin is red, calmodulin green, lysozyme blue, and LMPTP purple. Color transparency was adjusted to 0.5 for effective visualization of the compared datasets. **a** The Rosetta Ref15 distribution (gray) is compared to scoring with Rosetta Ref 15+ *hrf_dynamics* (color). **b** The distribution from scoring with Rosetta Ref15 (gray) is compared to top models when mover models are included in the distribution and scored with Rosetta Ref15 + *hrf_dynamics* (color).

Another metric of interest was the RMSD distribution of the top 1000 scoring models, as shown in Fig. 4a. Improvements in the percentage of the top-scoring models under 10.0 Å were seen for three of the four benchmark proteins when scoring with Rosetta Ref15 versus rescoring with *hrf_dynamics*. Calmodulin percentages stayed constant at 34.1% while lysozyme percentages increased from 11.9% to 12.4%, respectively, from Rosetta Ref15 to rescoring that included *hrf_dynamics*. While myoglobin improved from 99.7 to 100%, the average RMSD of the distribution improved from 4.62 Å with Rosetta Ref15 to 3.36 Å with rescoring.

Overall, the inclusion of *hrf_dynamics* with Rosetta scoring tended to improve the best scoring model quality, *P*_near_, and RMSD distributions of top-scoring models, indicating that the usage of HRPF data and dynamics-driven agreement enhanced Rosetta protein structure prediction.

### Rosetta mover ensemble combined with rescoring further improved RMSD of the lowest scoring model

Using the *hrf_dynamics* score term, which accounted for dynamics effects in its development, we saw an improvement of the RMSD of the best scoring model for all four benchmark proteins. However, we hypothesized that we could further improve the RMSD of the best scoring model for all four benchmark proteins by explicitly sampling protein flexibility around the best scoring structures. The top 20 scoring structures (when scored with both Ref15 and *hrf_dynamics*) for each protein were used to generate 30 models per structure with the Rosetta mover ensemble. The mover models were used to sample side-chain dynamics of ab initio models; no crystal structure was required to model the side-chain dynamics as the ab initio models were used as input. We hypothesized that this would sample side chain configurations that are in better agreement with the labeling data. The generated models were then scored with Ref15 and *hrf_dynamics*. As observed in Figs. 2c and 3c, the best scoring model RMSDs further improved for all four proteins when the mover protocol was explicitly applied to the top 20 scoring models. The best scoring mover model for calmodulin had an RMSD value of 7.24 Å, an improvement in RMSD from the best scoring model RMSD of 9.13 Å with rescoring alone. The best scoring model for lysozyme had an RMSD value of 4.37 Å, also an improvement from the best scoring model RMSD of 6.65 Å with rescoring alone. LMPTP’s best scoring model RMSD improved from 1.67 to 1.55 Å. While the best scoring model RMSD with rescoring alone was 4.85 Å for myoglobin, the best scoring mover model RMSD was 2.37 Å.

We then added the 600 mover models to our set of 20,000 ab initio structures for each of the four benchmark proteins, creating a set of 20,600 structures (Fig. 2c). The lowest scoring mover models were also the top-scoring model for the score versus RMSD distribution of all 20,600 structures, reaffirming our success with the mover set. Furthermore, we recalculated *P*_near_ values for the set of 20,600 structures. The *P*_near_ values increased for all four benchmark protein sets. LMPTP *P*_near_ value increased slightly from 0.66 with rescoring to 0.67 when movers were included in the distribution. Calmodulin and lysozyme also saw *P*_near_ improvements, from 0.07 to 0.14 for calmodulin and from 0.19 to 0.22 for lysozyme. Finally, myoglobin saw the largest improvement in *P*_near_, increasing from 0.29 to 0.56 with the inclusion of mover models in the distribution.

Moreover, we compared the RMSD of the top-scoring models including movers to the crystal structure via RMSD over SSEs. LMPTP RMSD_SSE_ stayed constant at 1.58 Å. Calmodulin RMSD_SSE_ of the best scoring model improved from 8.01 Å with the rescoring to 6.45 Å with mover models included in rescoring. The RMSD_SSE_ for myoglobin improved at each step from 4.67 Å with rescoring to 2.11 Å with movers included in rescoring. Lysozyme showed a notable improvement, with the RMSD_SSE_ of the best scoring model improving from 11.55 Å with Rosetta to 3.53 Å with the rescoring to 1.26 Å with movers included in rescoring. Again, the incorporation of the mover models allowed us to identify models with accurate atomic detail in secondary structure elements for three of the four benchmark proteins. This underscores the usefulness of HRPF data in protein structure determination.

We also investigated the RMSD frequency of the top 1000 scoring models from the set of 20,600 structures that included mover models, shown in Fig. 4b. The percentage of top-scoring models under 10.0 Å continued to increase for all four proteins, with calmodulin improving from 34.1% with rescoring to 39.8% rescoring with movers and lysozyme from 11.9 to 33.5%. The percentage of LMPTP models under 10.0 Å also increased from 49.8% with rescoring to 52.0% with mover models included in rescoring. While all myoglobin top models remained under 10.0 Å, the average RMSD of the top-scoring models further improved from 3.36 Å with rescoring alone to 3.16 Å with the inclusion of mover models in rescoring. The improvement of all four benchmark proteins exhibited for top-scoring model RMSD, *P*_near_ values, and RMSD distributions of the top 1,000 scoring models further demonstrated the potential of dynamics-driven HRPF modeling to obtain higher-quality models.

In addition to our benchmark set of four proteins with at least 15 labeled residues and residue-resolved PF data, we ventured to test our method on datasets that fell outside our benchmark set criteria. We used cytochrome C data that was employed in our previous work and identified an additional protein, cofilin, for which PF data was available. After generating 20,000 ab initio models per protein, we performed the *hrf_dynamics* rescoring protocol described above. Supplementary Fig. 7 includes the score versus RMSD plots for both additional proteins (Supplementary Fig. 7A, C), as well as the best scoring models aligned with the crystal structure (Supplementary Fig. 7B, D). The best scoring model RMSD for cytochrome C improved from 2.42 Å with Rosetta to 1.38 Å with Rosetta and *hrf_dynamics* combined; the cofilin best scoring model RMSD remained at 1.78 Å upon scoring with *hrf_dynamics*. 30 mover models were generated for each of the top 20 scoring structures and then scored with *hrf_dynamics* and included in the original ab initio model distribution. We found that the best scoring model RMSD for cytochrome C improved slightly to 1.37 Å, and the cofilin best scoring model RMSD improved to 1.52 Å. Despite the original datasets falling outside the benchmark criteria in place, the improvements for the additional proteins demonstrated that the *hrf_dynamics* term as well as the Rosetta mover ensemble are a powerful tool to predict protein structure from covalent labeling data, even for smaller numbers of labeled residues.

For further analysis of our benchmark set results, we compared our results to those in previously published work. For one of the proteins in our benchmark set, homology modeling and HRPF agreement analysis had been done previously in the pioneering work by Xie et. al. In this work, homology modeling was used to obtain models for lysozyme with RMSDs ranging from 1.2 to 4.6 Å^19^. In addition, it was possible to discriminate between models with backbone RMSD values greater than 4 Å and models with backbone RMSD values less than 3 Å for lysozyme^19^. As part of our ab initio modeling, we built models for lysozyme with RMSD values ranging from 4.24 to 28.14 Å. Thus, for lysozyme, the homology models presented by Xie and coworkers were of noticeably better quality than those presented in our ab initio work. To demonstrate that this discrepancy can be solely attributed to the use of homology modeling (versus ab initio modeling), we generated lysozyme homology models to analyze the HRPF agreement using our developed algorithm. We used similar templates for lysozyme as those used in ref. ^19^. In addition to building homology models for lysozyme, we also built models for myoglobin, for which data was also presented in ref. ^19^. but no homology models were built. We built homology models ranging from 0.50 to 1.82 Å for lysozyme and 0.72 to 2.88 Å for myoglobin. After rescoring and generation of mover models, we selected the best scoring model with RMSD 0.62 Å for lysozyme and 1.05 Å for myoglobin, indicating that our developed algorithm can successfully identify homology models showing atomic detail for these proteins. Our results and templates used are shown in Supplementary Fig. 8.

We also strove to assess our work based upon our own previous work that used neighbor counts and ab initio modeling. Both our current and previous work focused on the difficult cases requiring template-free modeling where the inclusion of covalent labeling data is most beneficial. While some of the benchmark proteins in our set are different from the previous set (benchmark of static HRPF modeling for myoglobin, calmodulin, lysozyme, and cytochrome C), we could compare the overall range of values between the two studies. For instance, we examined the difference between the best scoring model RMSD and the lowest RMSD model generated. On average, there was a difference of 2.40 Å between the best scoring model RMSD and lowest RMSD model in the previous work; we improved this to an average difference of 1.62 Å, suggesting that we are identifying models closer to the correct native structure. Moreover, in prior work, the *P*_near_ ranged from 1.17 × 10^−6^ to 0.38 after rescoring with the previous score term^20^. The *P*_near_ values in our current work ranged from 0.14 to 0.67 when movers were included and the 20,600 model set was rescored with *hrf_dynamics*. We also improved over previous work in terms of average RMSD over SSEs. The average RMSD_SSE_ for the top rescoring model to the crystal structure was 4.79 Å in the previous work. We surpassed this average with the top models from movers included in rescoring, yielding an average RMSD_SSE_ value of 2.85 Å. The improvements validated our efforts to incorporate dynamics into the prediction equation, suggesting that accounting for side-chain flexibility allowed us to exceed improvements seen in previous work. These improvements further stressed that the combination of HRPF data and side-chain flexibility can refine protein structure prediction and yield better model quality.

Hydroxyl radicals covalently label residues according to the residue’s solvent exposure. Correlations between exposure metrics of protein crystal structures and PFs have been demonstrated but are not perfect. We hypothesized that side-chain dynamics could account for some of the discrepancies between neighbor count and PF. This work has shown that incorporating side-chain flexibility as sampled by a Rosetta mover ensemble improved the NRMSE of neighbor counts and HRPF PFs, and this improvement can be capitalized upon for rescoring ab initio models. Our benchmark set, comprised of HRPF data for myoglobin, calmodulin, lysozyme, and LMPTP, was used to explore the effect of side-chain flexibility in HRPF-guided structure prediction. Overall, NRMSE values tended to decrease when flexibility was taken into account with MD or Rosetta movers. Because there is an over 170-fold decrease in run time with Rosetta movers compared to MD simulations, usage of Rosetta movers can potentially replace time-consuming MD simulations for the purpose of sampling side-chain exposure in HRPF-guided modeling, providing a less computationally expensive alternative. Based on these findings, we successfully implemented a Rosetta score term that showed a notable decrease of the RMSDs of the lowest-scoring ab initio models and increased the funnel-like metric *P*_near_ in benchmark tests. While our findings with MD and Rosetta did not have statistically significant implications in NRMSE, we noticed systematic, positive improvements in the downstream effects from using the prediction equation such as model selection and funnel-like quality of score versus RMSD distributions. RMSDs of best scoring ab initio models and *P*_near_ values improved, suggesting that accounting for protein flexibility when modeling HRPF data can improve model quality. Finally, the capability of the Rosetta mover ensemble was cemented by the improvement in the RMSD of lowest-scoring mover models generated from the top 20 scoring ab initio structures for all benchmark proteins. While impressive model generation and HRPF discrimination for homology models had been shown previously by Xie et al., the combination of HRPF data and sidechain flexibility sampled by the Rosetta mover ensemble has been demonstrated to yield results that are accurate to a level not before seen with covalent labeling-guided ab initio protein structure prediction. Furthermore, our work established HRPF as one of the prime experimental techniques in modeling protein structure from comparatively sparse experimental data. This is particularly important due to the ease with which HRPF data can be generated. In an attempt to make our algorithm widely accessible to the HRPF MS community, an in-depth tutorial describing the modeling protocol, along with necessary command lines, is provided as part of this manuscript in Supplementary Note 1.

In addition to incorporating the findings presented here into the ab initio protocol, future work will pursue the Rosetta mover ensemble in conjunction with other covalent labels to continue to improve Rosetta protein structure prediction. Future work will focus on investigations of the microenvironmental effects of covalent labels and aim to incorporate these effects into structure prediction^13^. Finally, in future work, we plan to explore long-timescale dynamics governing linked secondary or other higher-order structure perturbations using long MD simulations. We speculate that accounting for microsecond-timescale events has the potential to improve agreement with HRPF data for some proteins.

## Methods

### Benchmark set

Our benchmark set was comprised of four globular proteins for which hydroxyl radical PF data was available. We required each protein within our benchmark set to have at least 15 labeled residues since we demonstrated previously that a higher number of labeled residues strongly correlates with prediction accuracy^18^. We additionally required PF data to be residue-resolved. Myoglobin (PDB: 1YMB, 153 residues) and lysozyme (PDB: 2LYZ, 129 residues) data originated from work by Xie and coworkers^19^. PFs of 33 residues from myoglobin and 19 residues from lysozyme were extracted. Calmodulin (PDB: 1PRW, 148 residues) data was obtained from the manuscript of Kaur and colleagues^28^. PFs of 29 labeled residues were extracted. LMPTP (PDB: 5JNS, 152 residues) data was found in the work by Stanford et al.^29^ PF data for 26 labeled residues were extracted.

### Additional proteins examined

While not included in our benchmark set, we also examined two additional proteins. Cytochrome C (PDB: 2B4Z, 104 residues) data were obtained from ref. ^20^. PF values for 14 residues were available. Cofilin (PDB: 1CFY, 143 residues) data were extracted from Guan et al.^38^ Fragment-resolved data were available for 11 residues. Each labeled residue identified within the fragment was assigned the same PF as the fragment.

### Definitions of exposure metrics

PF, a metric originally introduced by Huang and coworkers, was used^30^. PF relates the experimentally determined labeling rate constant and amino acid-specific intrinsic reactivity. We have used the natural logarithm of the PF (lnPF, Eq. (1)) in this work^20^.

1 $${\mathrm{lnPF}} = {\mathrm{ln}}\frac{{R_i}}{{k_i}}$$

*R*_*i*_ represents the amino acid intrinsic reactivity for residue *i* while *k*_*i*_ represents the experimentally determined labeling rate constant for residue *i*.

Based on the improvement of the neighbor count with the cone method shown in our previous work, we continued to optimize the conical neighbor count^18^. The conical neighbor count was calculated as the sum of the products of the distance and angle contributions, with the angle contribution, A(Θ_*ij*_), shown in Eq. (2)^18^.

2 $$A\big( {{\mathrm{{\Theta} }}_{ij}} \big) = \frac{1}{{1 + {\mathrm{exp}}(2\pi * ({\mathrm{{\Theta} }}_{ij} - M))}}$$

Here, *2π* is the angle steepness value. *M* represents the angle midpoint value, which was optimized in this work. Angle midpoints of π/6, π/4, *π*/2, and *π* for conical neighbor count were calculated with the Rosetta *per_residue_solvent_exposure* application and subsequently correlated with lnPF for individual proteins in the benchmark set and all proteins combined. Normalized root means square error (NRMSE, Eq. (3)) and regression correlation, *R*^2^, were evaluated.

3 $${\mathrm{NRMSE}} = \frac{{\sqrt {\frac{{\mathop {\sum}\nolimits_{i = 1}^n {{\mathrm{diff}}_{\mathrm{i}}^2} }}{n}} }}{{\bar y}}$$

where *diff*_*i*_ represents the absolute difference between the observed neighbor count and the predicted neighbor count of residue *i*, while *n* represents the number of labeled residues. *ȳ* is the averaged of the observed neighbor counts.

### Determination of residues used in structure prediction

Nineteen of the 20 amino acids can be labeled by HRPF with varying levels of reactivity and reliability^30^. We eliminated cysteine and methionine from our evaluation because of the high reactivity of these residues^19^. In addition, it has been reported that residues such as serine, threonine, and aspartic acid can have unclear products during longer exposure, bringing into question their reliability and leading us to exclude them from consideration^16,17^. We focused on residues with sequential intrinsic reactivities, thus including W, Y, F, H, L, I, R, K, V, P, and E in our investigation^30^. While we included arginine in our analysis, arginine has previously been shown to potentially undergo deguanidination that results in a sizeable mass change^16^. We tested residue type combinations of at least three residue types so as to have an adequate number of data points. The residue combinations we examined included WYF, WYFH, WYFHL, WYFHLIR, WYFHLIRK, WYFLIRKV, WYFLIRKVP, and WYFLIRKVP. If a successful residue type combination contained arginine, we planned to further assess its reliability as a label; however, arginine was not included in our final residue subset. Conical neighbor counts were calculated for the benchmark set protein crystal structures. Labeled residues from the residue type combination tested were used to plot neighbor count versus PF using Matplotlib 3.1.2 and Python 3.7. NRMSE and *R*^2^ values were calculated based on the predicted and measured neighbor counts for the labeled residues of all proteins individually; values for all proteins together were calculated for all labeled residues in all proteins.

### MD simulations

To sample side-chain flexibility, the proteins in the benchmark set were prepared for MD simulations with Nanoscale Molecular Dynamics (NAMD)^31^. Structures were solvated with explicit TIP3P water molecules in a 14 Å water box and then neutralized during an ionization step with 0.15 M NaCl. With protein system restraints, the water molecules were minimized for 10,000 steps, and the proteins subsequently were minimized for 10,000 steps. An initial equilibration over 190,000 steps removed restraints. A final equilibration was executed for 10,000 steps. The MD simulations were performed using NAMD 2.12 with the CHARMM36 force field^39^. The simulations were performed at 310 K in the NPT ensemble with Langevin temperature and pressure dampening. The SHAKE algorithm was employed to constrain bonds with hydrogen, providing a 2 fs time step. Production runs were performed for 200 nanoseconds, or 100,000,000 timesteps, using one node with 28 processors per simulation on the Owens cluster of the Ohio Supercomputer Center^35^.

Structures were extracted from each saved simulation frame (every 2 ps). Conical neighbor counts were calculated for labeled residues of each frame and were averaged for a total simulation length of 200 ns. Neighbor counts from frames extracted every 2, 10, 20, 30, 50, 100, 500, or 1000 ps were averaged for the 200 ns simulation. The NRMSE of lnPF versus neighbor count was determined for each protein individually and for all proteins together using Python 3.7.

### Rosetta relaxation ensemble

A combination of Rosetta movers was employed to mimic the side chain flexibility sampled with MDs. A Rosetta XML script was implemented that combined the *NormalModeRelax* and *FastRelax* movers for the generation of 10, 20, 30, 40, 50, 100, 150, or 200 structures per protein in the benchmark set^32,33^.

The *NormalModeRelax* mover, which attempts multiple normal modes, was used with the Ref15 cartesian score function to relax and score poses. Cartesian normal mode was implemented. Five normal modes were explored with a mixture of modes used on 20 structures. A 2.0 Å perturbation was applied to backbone atoms, then a *FastRelax* was performed. The *FastRelax* mover was subsequently employed again with the Ref15 cartesian score function and with 25 *FastRelax* repeats performed per structure.

Conical neighbor counts were calculated for labeled residues of each generated structure, then the neighbor counts were averaged per labeled residue over all generated structures. The average neighbor count was correlated with PF, and the NRMSE value was assessed for 10, 20, 30, 40, 50, 100, 150, and 200 models per protein generated with the relaxation ensemble.

### Ab initio model generation

The Rosetta *AbInitio Relax* protocol was used to generate 20,000 models of each benchmark protein^40^. The protocol requires inputs of fragment libraries and native FASTA sequences^41^. The FASTA sequence of each protein was obtained from the respective PDB files and subsequently used by the Robetta server to extract fragment libraries for each protein^42^. No HRPF data were used during the fragment generation and ab initio model building. The models were scored with the Rosetta energy function (Ref15), and the crystal structure of each protein was supplied for RMSD calculations to the models. The Rosetta score was used to rank the generated models by their predicted agreement to the native protein structure.

In addition, some of the ab initio models generated were used to test the neighbor count prediction equation obtained from the relationship between lnPF and conical neighbor count averaged over 30 mover models. The top ten scoring ab initio models with RMSD values to the crystal structure greater than 10 Å for each benchmark protein were assembled, along with 10 top-scoring models with RMSD less than 5 Å from the best RMSD model generated. The observed conical neighbor counts were calculated using the *per_residue_solvent_exposure* application with an angle midpoint value of *π*/2. The predicted conical neighbor counts were calculated by substituting the experimental lnPF value of each labeled residue into the prediction equation determined from the Rosetta mover ensemble, as shown in Eq. (4).

4 $${\mathrm{Predicted}}\,{\mathrm{neighbor}}\,{\mathrm{count}} = 0.88\left( {{\mathrm{lnPF}}} \right) + 4.42$$

The resulting neighbor count is henceforth referred to as the predicted neighbor count. Delta values ranging from 0.5 to 4.5 (with an interval of 0.1) were used to determine the agreement of predicted values with observed values. The percentage and number of labeled residues that fell within the delta region were compared between top-scoring low RMSD models and top-scoring high RMSD models.

### Rescoring with *hrf_dynamics* and evaluation of ab initio models

Based on the dynamics-driven agreement between HRPF data and neighbor counts, a Rosetta score term, *hrf_dynamics*, was implemented to restore models. Each labeled residue’s predicted neighbor count was calculated by substituting lnPF into the equation relating neighbor count to experimental PF (Eq. (4)). The observed neighbor count was determined from the structure based on the product of the distance and angle contributions^18,20^. The absolute difference between the predicted and the observed neighbor count, |*diff*_*i*_|, was calculated for each labeled residue *i*. Based on the absolute value of the difference between observed and predicted neighbor counts, the *hrf_dynamics* score was calculated by summing up the per-residue contributions, as shown in Eq. (5)

5 $${\mathrm{hrf}}\_{\mathrm{dynamics}} = \mathop {\sum}\limits_i^n {\frac{{ - 1.0}}{{1.0 + {\mathrm{exp}}\left( {2.0\left( {\left| {{\mathrm{diff}}_{\mathrm{i}}} \right| - 3.5} \right)} \right)}}}$$

in which *n* represents the number of labeled residues, 2.0 is the steepness, and 3.5 is the delta value. By design, the per-residue *hrf_dynamics* score for a labeled residue *i* resulted in a value ranging from 0, complete disagreement with the predicted neighbor count, to −1, complete agreement.

The *hrf_dynamics* score term was used to score the 20,000 ab initio models generated per benchmark protein. The Rosetta total score was determined from a weighted sum of the *hrf_dynamics* term and the original Ref15 score

6 $${\mathrm{Total}}\,{\mathrm{Rosetta}}\,{\mathrm{score}} = \left( {12.0 \cdot {\mathrm{hrf}}\_{\mathrm{dynamics}}} \right) + {\mathrm{Ref}}15\,{\mathrm{score}}$$

Weight values from 1 through 20 were examined. Weight values larger or equal to 12.0 were found to consistently maximize improvement for the four benchmark proteins. An in-depth tutorial describing this process, along with necessary command lines, and an example data set can be found in Supplementary Note 1 and Supplementary Data 1. Ab initio models were ranked by score, with lower scores having a better ranking.

The performance of both Ref15 and the *hrf_dynamics* re-scored total score were analyzed by several metrics. First, since more native-like structures should have lower scores, the total Rosetta score was plotted against model RMSD, and the resulting distribution was analyzed. In an ideal scenario, the lowest-scoring model should have the lowest RMSD to the native. Best-scoring models were aligned to the native and visualized using PyMOL 2.0.6. We investigated the best-scoring model’s RMSD to the native structure to judge the effectiveness of the scoring methods. Secondly, we used the metric *P*_near_ as defined by Bhardwaj et al. and used it in our previous works^18,20,37^. The *P*_near_ was calculated using Eq. (7):

7 $${P}_{{\mathrm{near}}} = \frac{{\mathop {\sum}\nolimits_{m = 1}^N {{\mathrm{exp}}\left( { - \frac{{{\mathrm{rmsd}}_m^2}}{{\lambda ^2}}} \right){\mathrm{exp}}\left( { - \frac{{{\mathrm{score}}_m}}{{k_BT}}} \right)} }}{{\mathop {\sum}\nolimits_{m = 1}^N {{\mathrm{exp}}\left( { - \frac{{{\mathrm{score}}_m}}{{k_BT}}} \right)} }}$$

where *N* is the total number of models, *score*_*m*_ is the Rosetta score and *rmsd*_*m*_ is the RMSD of a particular model, *m*. *k*_*B*_*T*, which determines the funnel depth effect on *P*_near_, was used with a value of 1.0^18,20,37^. *λ*, a value that specifies which models are considered similar to the native, was retained at a value of 2.0 Å^18,20^. A *P*_near_ value of 1 indicates a perfect funnel-like shape, while a value of 0 indicates the lack of any funnel-like distribution. *P*_near_ was calculated using Python 3.7. Furthermore, we analyzed the RMSD over SSEs for each of the top scoring-models by extracting the residues involved in SSEs from the PDB file. We truncated the structural files to only include these residues, then calculated the RMSD_SSE_ of the SSE models to the crystal structure with only SSE residues. Finally, we compared the number of top 1000 scoring models with RMSD below 10.0 Å. This comparison was performed to determine how well *hrf_dynamics* improved model quality over Rosetta Ref15.

### Generation and evaluation of mover models from top-scoring ab initio structures

In an attempt to further improve structure prediction by explicitly modeling side-chain flexibility, the top 20 scoring ab initio structures from each benchmark protein were then used to generate models using the Rosetta mover ensemble. The mover ensemble used the same settings as reported in the Rosetta Relaxation Ensemble section, in which the *NormalModeRelax* mover was used with the Ref15 cartesian score function to relax and score poses. Using cartesian normal mode, five normal modes were explored with a mixture of modes used on 20 structures. After a 2.0 Å perturbation was applied to backbone atoms, *FastRelax* was performed. The *FastRelax* mover using the Ref15 cartesian score function was used to perform 25 *FastRelax* repeats per structure. 30 mover models were generated for each of the 20 top scoring structures. The 600 resulting models per protein were scored with both Ref15 and *hrf_dynamics* (Eq. (5)) to obtain a total score (Eq. (6)). The 600 mover models were added to the 20,000 ab initio models, and the resulting 20,600 models were scored with Rosetta and *hrf_dynamics*. The same metrics used to evaluate performance, including the RMSD of the best scoring model, the RMSD over SSEs (RMSD_SSE_) of the best scoring model, the *P*_near_ (Eq. (7)), and the percentage of the top 1,000 scoring models under 10.0 Å, were used to determine if there was an improvement in model quality over both Rosetta and rescoring with *hrf_dynamics*.

### Rosetta homology model generation and evaluation with *hrf_dynamics* and mover models from top-scoring structures

Homology models for myoglobin and lysozyme were generated using the Rosetta Comparative Modeling protocol^43^. Multiple templates (Supplementary Fig. 8C) with high sequence coverage (95–100%) and varying sequence identities, ranging from 99 to 29% for myoglobin and from 99 to 37% for lysozyme, were used for model generation. Three thousand models were generated for each protein. Homology models were relaxed with the Rosetta Relax application and then scored with the Rosetta Ref15 scoring function^33^. RMSD values were calculated for the respective crystal structures. Models were rescored with the *hrf_dynamics* score term. After ranking the models by score, the top 20 scoring models were used as inputs for the Rosetta mover ensemble, using the same settings as reported in the Rosetta Relaxation Ensemble section and the previous section^32,33^. Thirty models were generated for each top-scoring structure. The 600 mover models were included in the ab initio model distribution, and the best scoring model RMSD was identified.

### Reporting summary

Further information on research design is available in the Nature Research Reporting Summary linked to this article.

## Supplementary information

Supplementary Information

Description of Additional Supplementary Files

Supplementary Data 1

Reporting Summary

## Data Availability

The authors declare that all data supporting the findings of this study are available within the paper and its Supplementary Information. The utilized protection factors can be found in refs. ^19,28,29^ for benchmark proteins and refs. ^20,38^ for additional proteins examined. Crystal structures for the four benchmark proteins are available on the Protein Data Bank with accession codes 1YMB, 1PRW, 2LYZ, and 5JNS. The accession codes for the additional proteins analyzed are 2B4Z and 1CFY. An example data set containing 2000 ab initio models and 600 mover models for myoglobin is provided in Supplementary Data 1.

## Code availability

The *hrf_dynamics* score term and Rosetta mover ensemble are available for free to academic users through the Rosetta software suite at https://www.rosettacommons.org/software/.
