# Supplementary material for: Accurate protein structure prediction with hydroxyl radical protein footprinting data
Source: Nat Commun. 2021 Jan 12;12:341. doi: 10.1038/s41467-020-20549-7 (PMC7804018; doi:10.1038/s41467-020-20549-7)
Supplement: Supplementary file 1 — Supplementary Information [file 41467_2020_20549_MOESM1_ESM.pdf]

Supplementary Information

Accurate Protein Structure Prediction with Hydroxyl Radical Protein Footprinting Data

Sarah E. Biehn[1] and Steffen Lindert[1*]

[1]Department of Chemistry and Biochemistry, Ohio State University, Columbus, OH 43210

*Correspondence to:
Department of Chemistry and Biochemistry, Ohio State University
2114 Newman & Wolfrom Laboratory, 100 W. 18th Avenue, Columbus, OH 43210
614-292- 8284 (office), 614-292-1685 (fax)
lindert.1@osu.edu

Protein(s)	PDB ID	Angle midpoint	NRMSE from lnPF vs. conical NC	R^2 from lnPF vs. conical NC, steepness 2π
myoglobin	1YMB	π/6	0.46	0.17
		π/4	0.36	0.19
		π/2	0.12	0.61
		π	0.13	0.52
calmodulin	1PRW	π/6	0.42	0.42
		π/4	0.36	0.41
		π/2	0.17	0.44
		π	0.09	0.21
lysozyme	2LYZ	π/6	0.31	0.10
		π/4	0.30	0.07
		π/2	0.19	0.08
		π	0.17	0.00
LMPTP	5JNS	π/6	0.39	0.67
		π/4	0.37	0.65
		π/2	0.33	0.48
		π	0.27	0.02
all	n/a	π/6	0.44	0.31
		π/4	0.39	0.29
		π/2	0.24	0.30
		π	0.19	0.05

Supplementary Table 1.
Summary of NRMSE and R^2 values for lnPF versus neighbor count calculated with various angle midpoint values using residues WYFHL for each protein within the benchmark set and for all proteins combined.

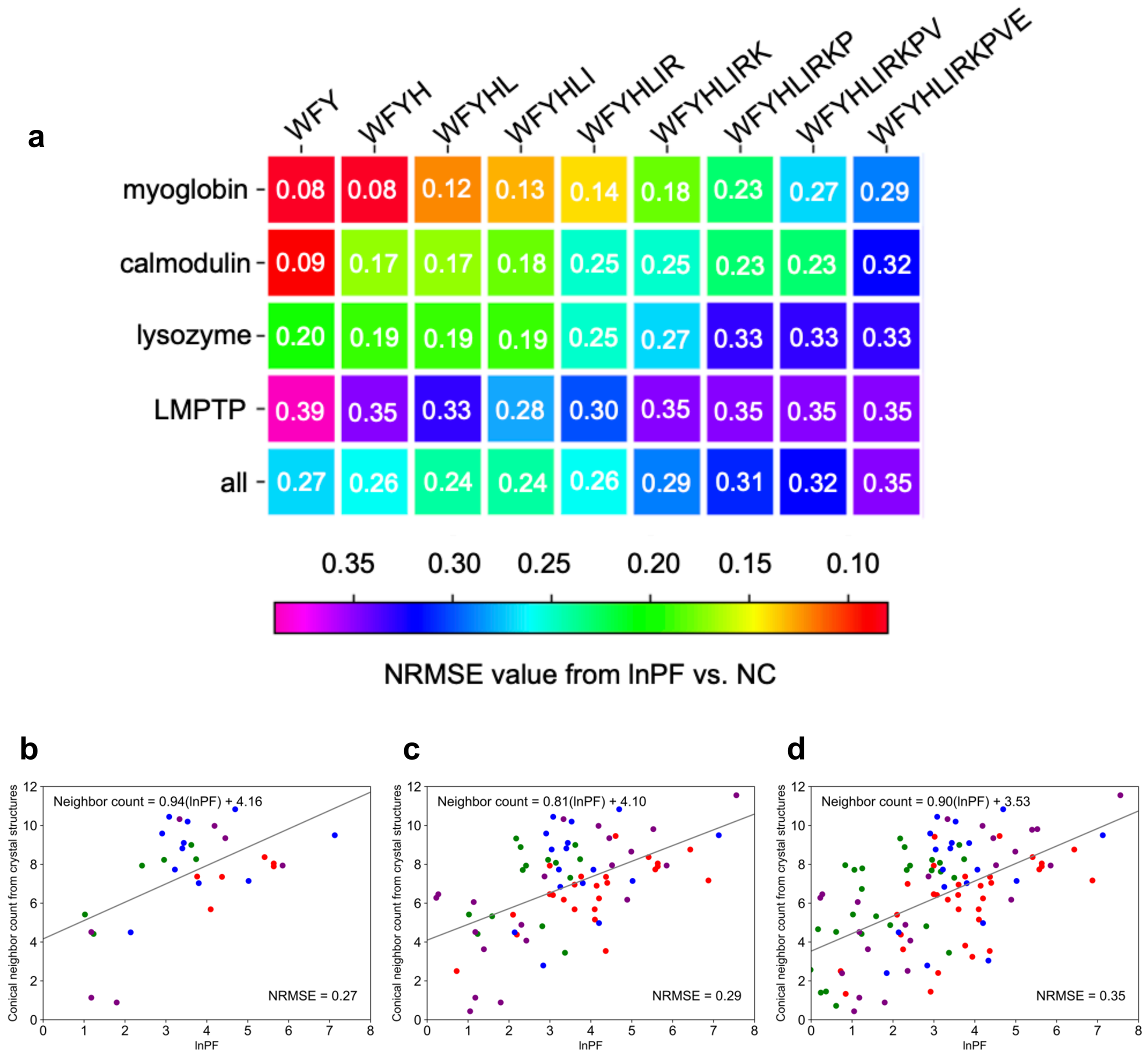

Supplementary Figure 1.

a, Heatmap summary of NRMSE values from conical neighbor count and lnPF for individual and all proteins when calculating neighbor count with various residue type combinations using the proteins' crystal structures.

lnPF versus conical neighbor count for all proteins when using residue types **b,** WYF, **c,** WYFHLIRK, and **d,** WYFHLIRKPV. Myoglobin labeled residues are red, calmodulin green, lysozyme blue, and LMPTP purple.

		myoglobin	calmodulin	lysozyme	LMPTP	all
	Crystal	0.12	0.17	0.19	0.33	0.24
200 ns MD simulation	every 2 ps	0.14	0.17	0.13	0.33	0.23
	every 30 ps	0.14	0.17	0.13	0.33	0.23
	every 50 ps	0.14	0.17	0.13	0.33	0.23
	every 100 ps	0.14	0.17	0.13	0.33	0.23
	every 500 ps	0.14	0.17	0.13	0.33	0.23
	every 1000 ps	0.14	0.17	0.13	0.22	0.23
# Rosetta mover models	10 structures	0.12	0.20	0.17	0.34	0.24
	20 structures	0.12	0.20	0.17	0.33	0.24
	30 structures	0.11	0.19	0.17	0.33	0.23
	40 structures	0.12	0.19	0.17	0.33	0.23
	50 structures	0.12	0.19	0.17	0.33	0.24
	100 structures	0.12	0.19	0.17	0.33	0.24
	150 structures	0.11	0.19	0.17	0.33	0.24
	200 structures	0.12	0.19	0.18	0.33	0.24

Supplementary Table 2.
NRMSE values calculated from lnPF versus crystal structure conical neighbor count, averaged neighbor count from different time points over 200 ns MD simulation, or averaged neighbor count from different numbers of Rosetta mover models generated for four benchmark proteins and all proteins combined.

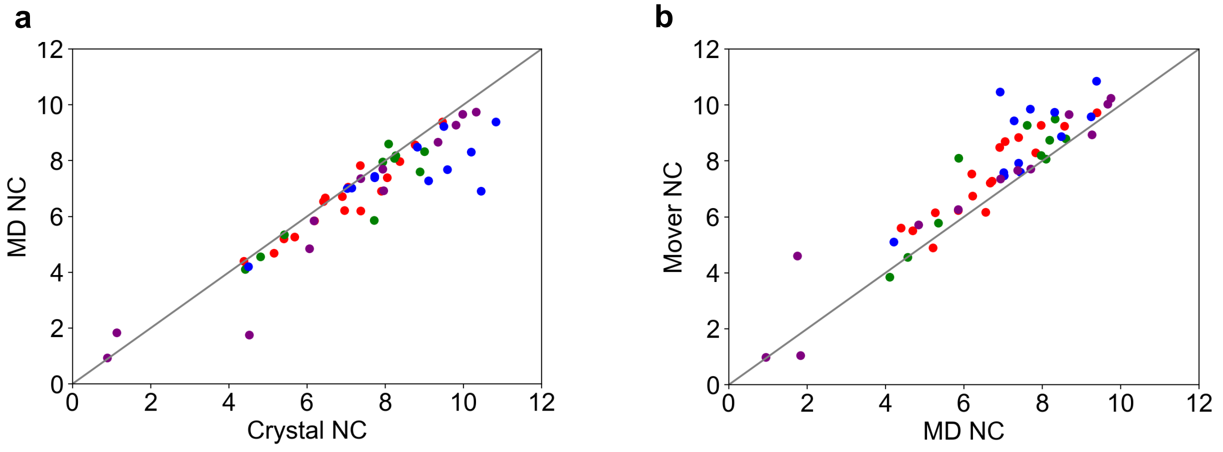

Supplementary Figure 2.
Comparison of conical neighbor counts from residues WYFHL when calculated **a**, from crystal structures and MD frames and **b**, from MD frames and mover models.

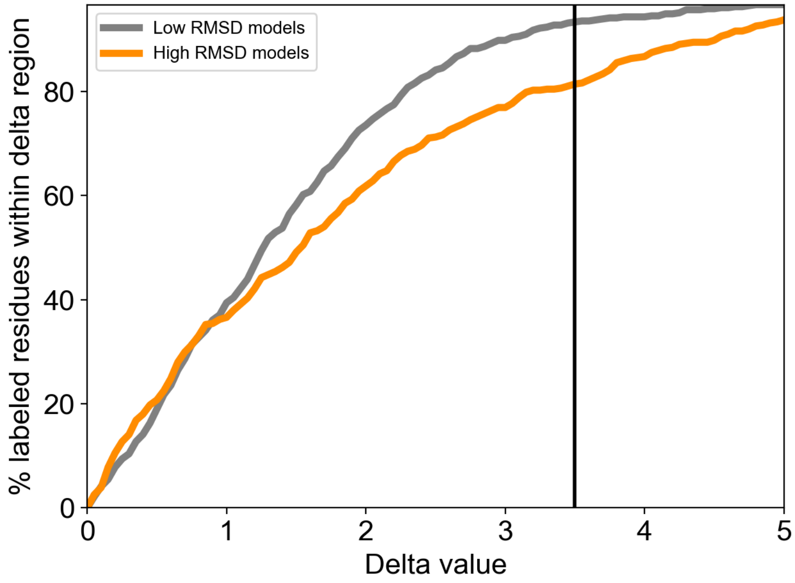

Supplementary Figure 3.
Percentage of labeled residues that fell within the delta region for various delta values. The percentage of labeled residues that fell within the delta region was compared between the top scoring low RMSD models (grey) and the top scoring high RMSD models (orange). The vertical line at delta = 3.5 indicates the delta value that was used in the *hrf_dynamics* score term.

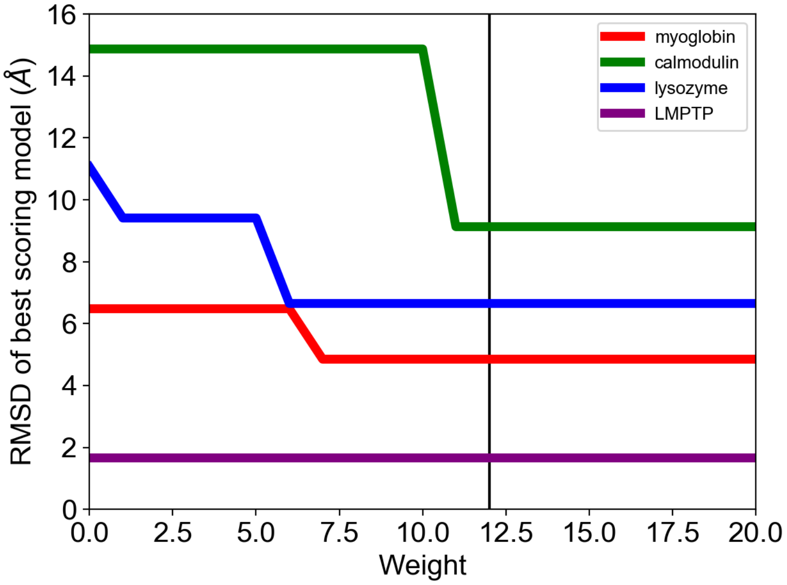

Supplementary Figure 4.
RMSD of the best scoring model after rescoring with *hrf_dynamics* at various weights for each protein's ab initio models. The vertical line at 12.0 indicates the weight used for our implementation of *hrf_dynamics*.

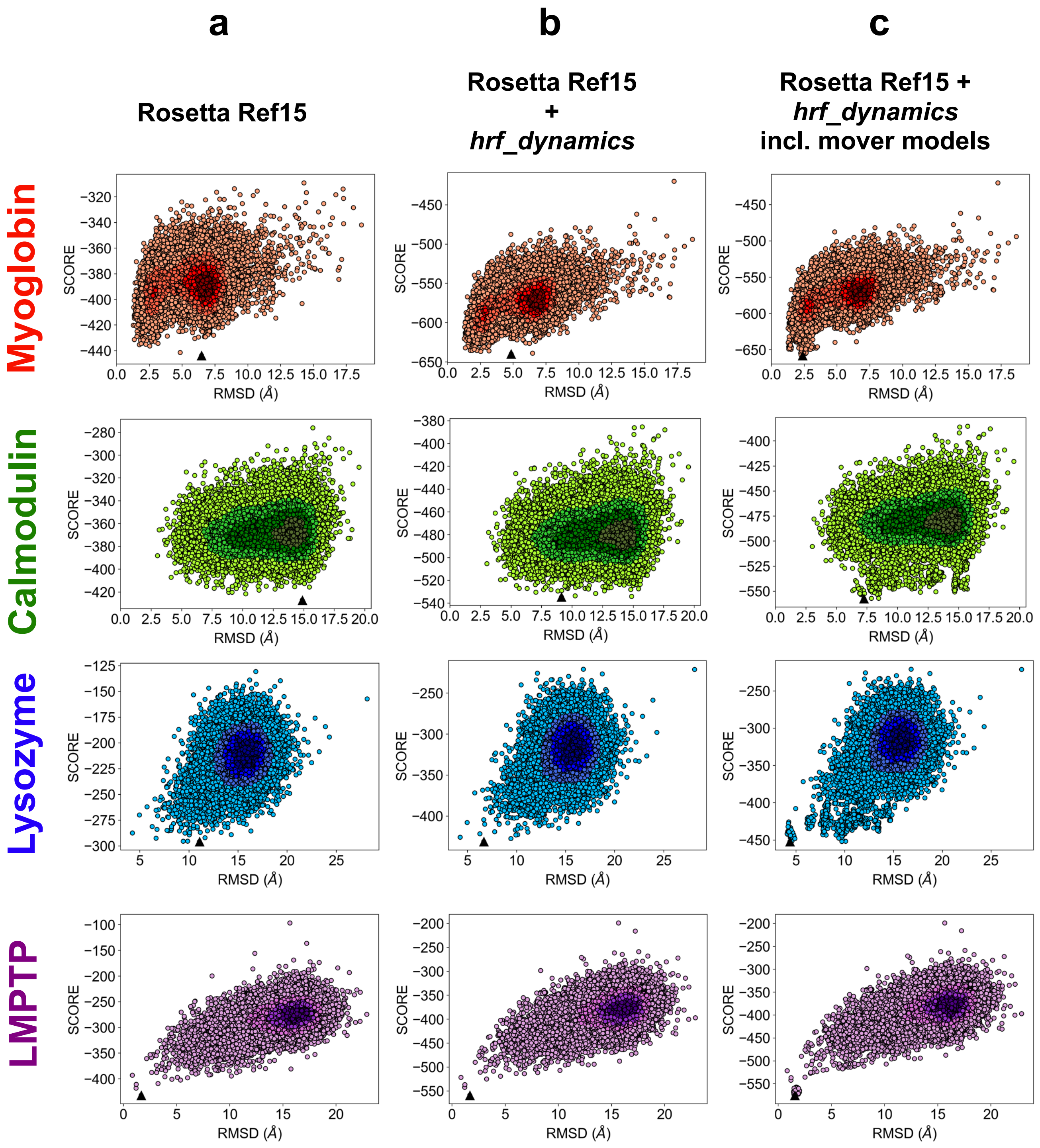

Supplementary Figure 5.

Score versus RMSD to the crystal structure density scatter plots for 20,000 ab initio models generated for each of the four benchmark proteins. Less dense regions are lighter in color while more dense regions are darker in color. **a**, Rosetta Ref15 score versus RMSD. **b**, Rosetta Ref15 + *hrf_dynamics* total score versus RMSD. **c**, Rosetta Ref15 +

hrf_dynamics total score versus RMSD, including the 30 mover models generated per top 20 scoring models.

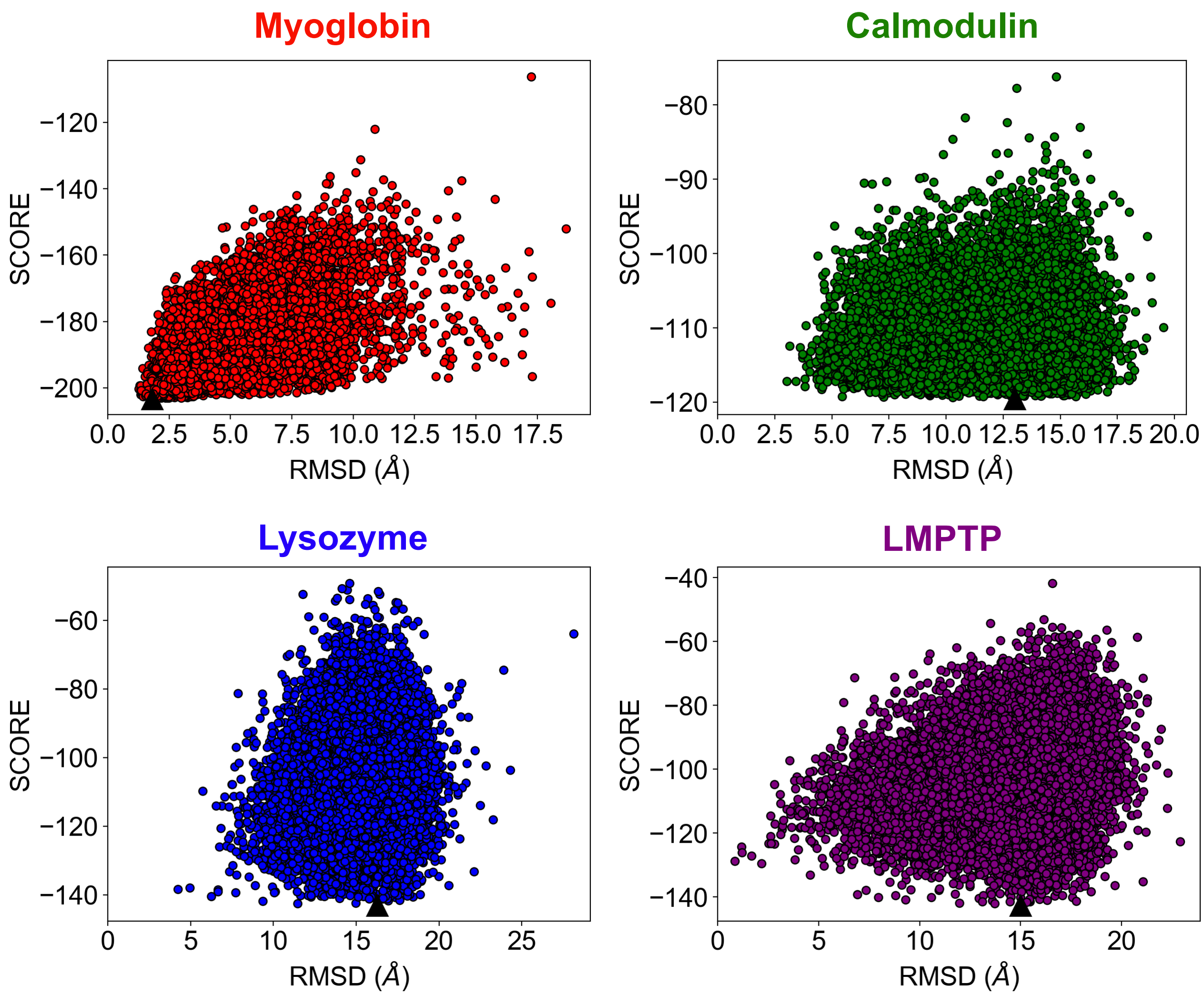

Supplementary Figure 6.
hrf_dynamics score vs. RMSD to the crystal structure for 20,000 ab initio models generated for myoglobin (red), calmodulin (green), lysozyme (blue), and LMPTP (purple). The top scoring model is marked by a black triangle.

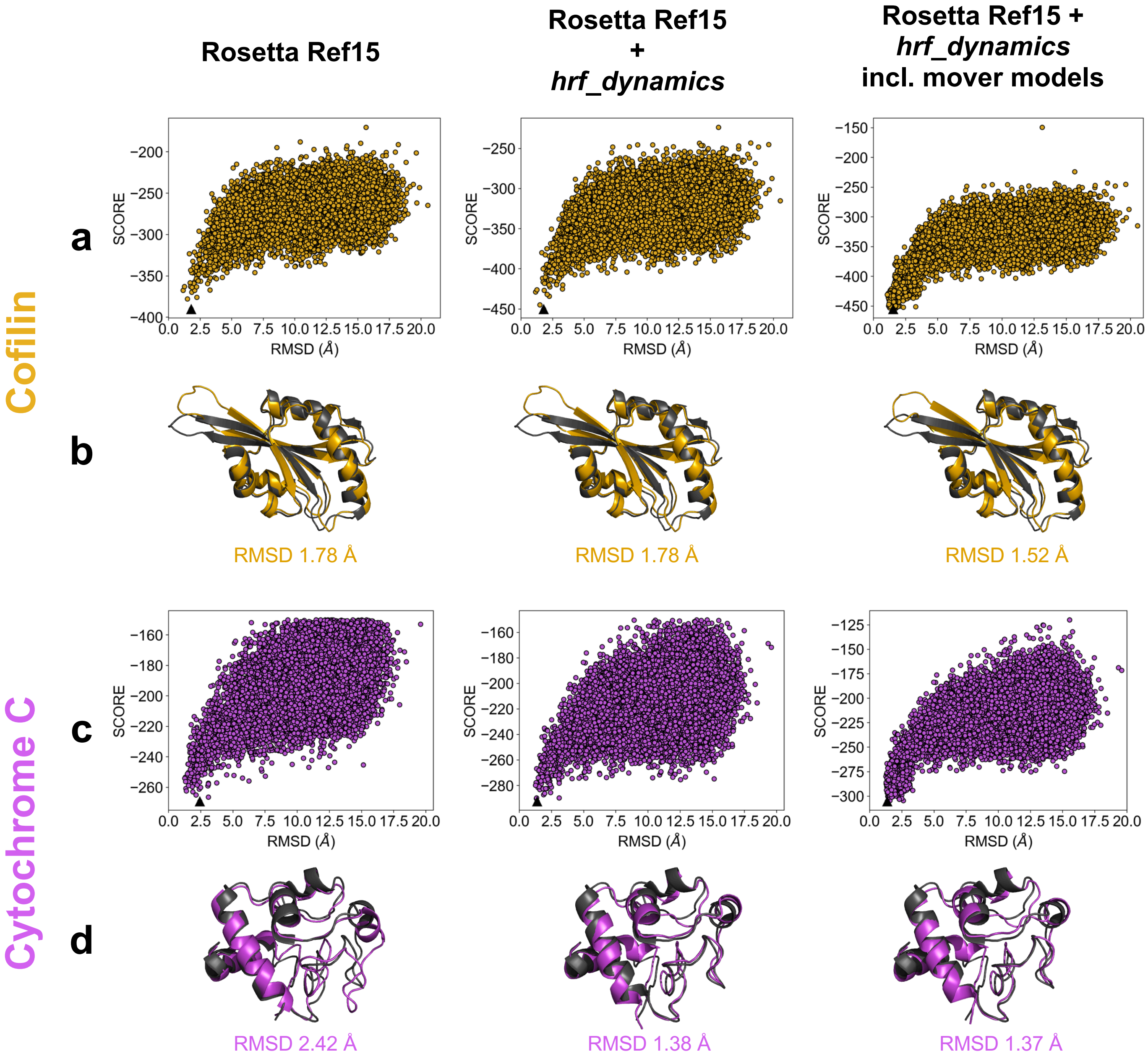

Supplementary Figure 7.
Score versus RMSD to the crystal structure for 20,000 ab initio models and crystal structures (dark grey) aligned with best scoring models (color) for additional proteins when scored with Rosetta Ref15, Rosetta Ref15 + *hrf_dynamics*, and Rosetta Ref15 + *hrf_dynamics* including 600 mover models. **a**, score versus RMSD for cofilin. **b**, crystal structure (grey) is aligned with best scoring model (gold). **c**, score versus RMSD for cytochrome C. **d**, crystal structure (grey) is aligned with best scoring model (orchid).

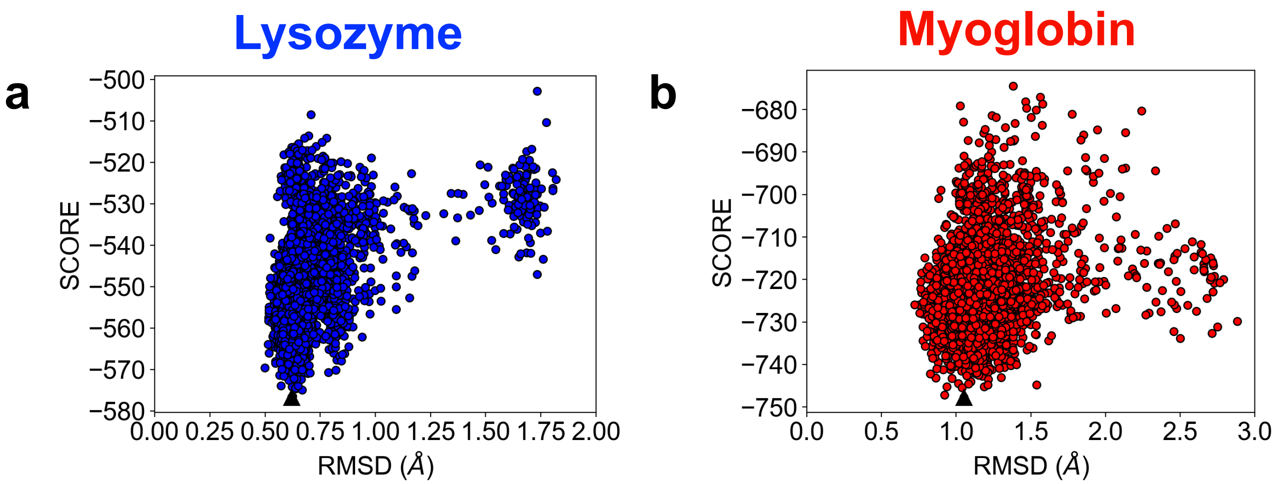

c

Target Protein	Template PDB ID	Sequence Coverage	Sequence Identity
lysozyme	1LZE	100%	99.2%
	2GV0	100%	69.0%
	2Z2E	99%	50.8%
	2BQJ	98%	59.8%
	4L41	95%	36.9%
myoglobin	1NZ3	100%	98.7%
	1LHS	100%	68.4%
	5OJC	99%	82.8%
	1MYT	96%	44.9%
	3AG0	96%	28.7%

Supplementary Figure 8.
Homology modeling for myoglobin and lysozyme. Score versus RMSD to the crystal structure for 3,600 homology models relaxed and scored with Ref15 + *hrf_dynamics* including the 30 mover models generated per top 20 scoring homology models for **a**, lysozyme (blue) and **b**, myoglobin (red). The top scoring model is marked by a black triangle. **c**, Sequence coverage and identity of templates used for homology modeling of the two proteins. Sequence coverage was maintained between 95-100% while sequence identity was varied from 99% to 37% for lysozyme and 99% to 29% for myoglobin.

Supplementary Note 1: Tutorials

Terminal commands are formatted in `fixed width`.
Example files are located in the `hrf_dynamics_tutorial` directory.

Tutorial 1: Using Rosetta to predict protein structure and rescoring with *hrf_dynamics*

This tutorial was written using a protein that has an available crystal structure in the PDB; however, **a known structure is not required to run this protocol**. The structure was used here to demonstrate the RMSD calculations performed in the manuscript, but these are not required for model generation.

1. Create a new directory for file inputs and outputs, then move into the new directory.
   ```
   > mkdir tutorial_one
   > cd tutorial_one
   ```
2. Fetch the crystal structure of myoglobin from http://www.rcsb.org/structure/1ymb.
   a. Download the file in PDB format.
   b. Move the downloaded PDB file into the `tutorial_one` directory.
   ```
   > cd Downloads
   > mv 1ymb.pdb /path/to/tutorial_one
   ```
3. PDB files often contain excess information, such as water molecules, experimental structure determination information, additional molecules, etc. The structure should be prepared for use with Rosetta by using the clean_pdb.py script that will remove the excess data. Execute the script with the specified structure and chain of interest.
   ```
   > python ~/Rosetta/tools/protein_tools/scripts/clean_pdb.py 1ymb.pdb A
   ```
4. Use Robetta to generate 3mer and 9mer fragments for *ab initio* structure generation.
   a. Visit the Robetta fragment generation server at https://robetta.bakerlab.org. Account registration is required for fragment generation.
   b. Use the Structure Prediction section to upload the appropriate PDB and FASTA files.
      i. For proteins with unsolved structures, primary sequences can be identified with UniProt.
         1. Visit the UniProt website: https://www.uniprot.org/
         2. Using search bar, search for protein of interest.
         3. Select the best option based on the organism from which the protein originates.
         4. Download the FASTA, which is found under the Sequence section. This file should be uploaded to Robetta.
   c. Upon job completion, download the fragment files, `aat000_03_05.200_v1_3` and `aat000_09_05.200_v1_3` files, to the `tutorial_one` directory.
      i. Rename the files:
      ```
      > mv aat000_03_05.200_v1_3 1ymb_frags3
      ```

```
> mv aat000_09_05.200_v1_3 1ymb_frags9
```

5. Create a flags file and save the newly generated file as `1ymb_abinitio_flags`. Note that it is important to preserve the indentation and spacing shown here:
```
-abinitio
  -relax
-in
  -file
    -fasta 1ymb_A.fasta
    -frag3 1ymb_frags3
    -frag9 1ymb_frags9
    -native 1ymb_A.pdb
-out
  -pdb
  -file
    -silent 1ymb_abinitio_silent.out
    -scorefile 1ymb_abinitio_scores.sc
-nstruct 10
```
   a. The `abinitio` and `relax` flags specify usage of the *AbinitioRelax* application.
   b. The `in` flag specifies the FASTA sequence file, the 3mer fragment file, the 9mer fragment file, and the crystal structure file (used only for RMSD calculation).
   c. Finally, the `out` flags are used to specify that a PDB structure file should be an output, as well as a score file that contains the Rosetta score and RMSD of the generated structure to the native.
6. Run the *AbinitioRelax* command in the terminal window:
```
> ~/Rosetta/main/source/bin/AbinitioRelax.linuxgccrelease
-database ~/Rosetta/main/database @1ymb_abinitio_flags
```
   a. The job should take 10-20 minutes if no errors occur.
      i. This tutorial only generates 10 *ab initio* models. For structure prediction, it is best practice to generate tens of thousands of models.
7. Prepare the input file for rescoring with *hrf_dynamics*.
   a. The score term requires an input file with the residue number in the first column and the lnPF in the second column. Create `lnPF_1ymb.txt` and add the protection factor data for labeled residues W, F, Y, H, and L to the file:
```
#Residue number and lnPF for myoglobin (1ymb)
7    4.094345
11   4.098900
14   4.370598
24   6.429719
…
```
   b. Note that the first line of the file will not be read by Rosetta and should be used as a header describing the contents of each column.
   c. Ensure that labeled residue numbers match the numbering in the PDB structure.

8. Create a flags file, `1ymb_hrf_flags`, for rescoring with the *score* application and *hrf_dynamics:*
```
-score
  -hrf_dynamics_input lnPF_1ymb.txt
  -weights hrf_dynamics.wts
```
   a. The `score` flag specifies the input file containing the residue number and lnPF values and indicates that the *hrf_dynamics* weights should be used.
9. Run the *score* application on one model:
```
> ~/Rosetta/main/source/bin/score.linuxgccrelease -database ~/Rosetta/main/database -in:file:s S_000001.pdb @1ymb_hrf_flags -out:file:scorefile 1ymb_hrf_rescores.sc
```
   a. Since all models need to be rescored, it is important to run the command for all models generated.
10. Analyze the results from the score file. This can be accomplished by examining the score file outputs.
    a. `1ymb_abinitio_scores.sc`
       i. Find the `score`, `rms`, and `description` columns. The `score` column provides the Rosetta Ref15 score. The `rms` column includes the RMSD to the native. The `description` column supplies the model name.
    b. `1ymb_hrf_rescores.sc`
       i. Find the `score` and `description` columns. The `score`, in this case, is the score of *hrf_dynamics.* In order to calculate the total score, the Ref15 score should be added to the *hrf_dynamics* score. The `description`, the structure name, should be used to add the appropriate scores for the same structure.

Tutorial 2: Generating Rosetta mover models from *ab initio* structures
Because Tutorial 1 generates only 10 *ab initio* structures, it is relatively easy to use the mover set on all of the *ab initio* models. Should tens of thousands of models be generated, it is recommended to use the mover script with a subset of top scoring models. The work in the manuscript used the movers with the top 20 scoring structures.

1. Create a new working directory in the home directory:
```
> mkdir tutorial_two
```
2. Copy the *ab initio* structures, the flags file, and the cleaned crystal structure from **Tutorial 1** into the new directory then move into the new working directory:
```
> cd tutorial_one
> cp S*pdb 1ymb_hrf_flags 1ymb_A.pdb ../tutorial_two
> cd ../tutorial_two
```
3. Next, run the Rosetta mover ensemble on all the *ab initio* structures:
```
> ~/Rosetta/main/source/bin/rosetta_scripts.linuxgccrelease -s S_000001.pdb -nstruct 30 -parser:protocol ~/Rosetta/main/source/scripts/rosetta_scripts/public/flexibility/nma_relax.xml -overwrite
```

a. `~/Rosetta/main/source/bin/rosetta_scripts.linuxgccrelease` calls the program to run the Rosetta script. Note that if an operating system other than Linux is being used, the application name will change based on the operating system.
b. `-s` tells Rosetta the name of the structure to use for model generation.
c. `-nstruct` controls the number of models generated with the mover ensemble. The `nstruct` flag indicates that 30 models should be generated per *ab initio* structure.
d. `-parser:protocol` flag is used to indicate that Rosetta mover ensemble script is running.

4. After the structures have been generated, rescore the mover models both with the *score* application and with *hrf_dynamics* for each model generated:

```
> ~/Rosetta/main/source/bin/score.linuxgccrelease -database
~/Rosetta/main/database -in:file:s S_000001_0001.pdb -
in:file:native 1ymb_A.pdb -out:file:scorefile 1ymb_rosetta-
.sc
> ~/Rosetta/main/source/bin/score.linuxgccrelease -database
~/Rosetta/main/database -in:file:s S_000001_0001.pdb
@1ymb_hrf_flags -out:file:scorefile 1ymb_hrfdynamics.sc
```

   a. A native structure is not required to calculate the score. Simply remove the `-in:file:native 1ymb_A.pdb` portion of the command if there is not a native structure.

5. Analyze the results from the score files:
   a. Extract the *score*, *rms*, and *description* entries from the `1ymb_rosetta.sc` file
      i. The *score* represents the Rosetta score alone, the *rms* is the RMSD to the native, and the *description* is the name of the structure.
      ii. If RMSD to a native was not calculated, the *rms* entry should not be extracted.
   b. Extract the *score* and *description* entries from the `1ymb_hrfdynamics.sc` file
      i. The *score* represents the *hrf_dynamics* score alone, and the *description* is the structure name.
   c. Sum the *score* and the *hrf_dynamics score* for each model to determine the model’s total score.
   d. If RMSD was calculated, a total score versus RMSD plot can also be generated.
