# Supplementary material for: Accurate protein structure prediction with hydroxyl radical protein footprinting data
Source: Nat Commun. 2021 Jan 12;12:341. doi: 10.1038/s41467-020-20549-7 (PMC7804018; doi:10.1038/s41467-020-20549-7)
Supplement: Supplementary file 2 — Description of Additional Supplementary Files [file 41467_2020_20549_MOESM2_ESM.pdf]

Description of Additional Supplementary Files

Supplementary Data 1

Supplementary Data 1 file contents:

1. **README.txt**: file that describes the code availability within Rosetta and an overview of file contents.
2. **example_data/1ymb_A.pdb**: structural coordinate file for chain A of myoglobin crystal structure 1YMB. Can be used in Tutorial 1 Step 9 and Tutorial 2 Step 4 for RMSD calculations.
3. **example_data/1ymb_hrf_flags**: flags file to be used when rescoring models with *hrf_dynamics* score term. (Tutorial 1 Step 9, Tutorial 2 Step 4)
4. **example_data/lnPF_1ymb.txt**: file containing labeled residue numbers and protection factor data to be used when rescoring models with the *hrf_dynamics* score term. (Tutorial 1 Step 9, Tutorial 2 Step 4)
5. **example_data/AbInitioStructures.zip**: 2,000 ab initio models generated for myoglobin. Can be used to rescore with the *hrf_dynamics* score term in Tutorial 1 Step 9.
6. **example_data/MoverModelsFromTop20.zip**: 600 mover models generated from the top twenty scoring ab initio models. Can be used to rescore with the *hrf_dynamics* score term in Tutorial 2 Step 4.
