# Supplementary material for: Accurate protein structure prediction with hydroxyl radical protein footprinting data
Source: Nat Commun. 2021 Jan 12;12:341. doi: 10.1038/s41467-020-20549-7 (PMC7804018; doi:10.1038/s41467-020-20549-7)
Supplement: Supplementary file 4 — Reporting Summary [file 41467_2020_20549_MOESM4_ESM.pdf]

nature research

Corresponding author(s): Steffen Lindert

Last updated by author(s): 12/07/2020

Reporting Summary

Nature Research wishes to improve the reproducibility of the work that we publish. This form provides structure for consistency and transparency in reporting. For further information on Nature Research policies, see our Editorial Policies and the Editorial Policy Checklist.

Statistics

For all statistical analyses, confirm that the following items are present in the figure legend, table legend, main text, or Methods section.

n/a	Confirmed	
☒	☐	The exact sample size (n) for each experimental group/condition, given as a discrete number and unit of measurement
☒	☐	A statement on whether measurements were taken from distinct samples or whether the same sample was measured repeatedly
☒	☐	The statistical test(s) used AND whether they are one- or two-sided<br>*Only common tests should be described solely by name; describe more complex techniques in the Methods section.*
☒	☐	A description of all covariates tested
☒	☐	A description of any assumptions or corrections, such as tests of normality and adjustment for multiple comparisons
☒	☐	A full description of the statistical parameters including central tendency (e.g. means) or other basic estimates (e.g. regression coefficient) AND variation (e.g. standard deviation) or associated estimates of uncertainty (e.g. confidence intervals)
☒	☐	For null hypothesis testing, the test statistic (e.g. F, t, r) with confidence intervals, effect sizes, degrees of freedom and P value noted<br>*Give P values as exact values whenever suitable.*
☒	☐	For Bayesian analysis, information on the choice of priors and Markov chain Monte Carlo settings
☒	☐	For hierarchical and complex designs, identification of the appropriate level for tests and full reporting of outcomes
☒	☐	Estimates of effect sizes (e.g. Cohen's d, Pearson's r), indicating how they were calculated

Our web collection on statistics for biologists contains articles on many of the points above.

Software and code

Policy information about availability of computer code

Data collection	The hrf_dynamics score term and Rosetta mover ensemble, nma_relax.xml, are freely available to academic users through the Rosetta Software Suite at https://www.rosettacommons.org/software.<br>Molecular dynamics simulations were performed with NAMD v2.12 using the CHARMM36 force field with SHAKE algorithm.
Data analysis	Python v.3.7.3, Matplotlib v.3.1.2, PyMOL v.2.0.6

For manuscripts utilizing custom algorithms or software that are central to the research but not yet described in published literature, software must be made available to editors and reviewers. We strongly encourage code deposition in a community repository (e.g. GitHub). See the Nature Research guidelines for submitting code & software for further information.

Data

Policy information about availability of data

All manuscripts must include a data availability statement. This statement should provide the following information, where applicable:

- Accession codes, unique identifiers, or web links for publicly available datasets
- A list of figures that have associated raw data
- A description of any restrictions on data availability

The authors declare that all data supporting the findings of this study are available within the paper and its supplementary information. The utilized protection factors can be found in references 19, 28, and 29 for benchmark proteins and references 20 and 38 for additional proteins examined. Crystal structures for the four benchmark proteins are available on the PDB with accession codes 1YMB, 1PRW, 2LYZ, and 5JNS. The accession codes for the additional proteins analyzed are 2B4Z and 1CFY. An example data set containing 2,000 ab initio models and 600 mover models for myoglobin is provided in Supplementary Data 1.

Field-specific reporting

Please select the one below that is the best fit for your research. If you are not sure, read the appropriate sections before making your selection.

- [x] Life sciences
- [ ] Behavioural & social sciences
- [ ] Ecological, evolutionary & environmental sciences

For a reference copy of the document with all sections, see nature.com/documents/nr-reporting-summary-flat.pdf

Life sciences study design

All studies must disclose on these points even when the disclosure is negative.

Sample size	No sample size calculation was performed. Data was extracted from previously published literature. Benchmark set proteins were chosen based on the availability of labeling data for at least 15 residues.
Data exclusions	No data were excluded.
Replication	Since Rosetta is Monte Carlo, model generation is not reproducible. However, the actual rescoring is reproducible once models have been generated. We have provided a set of 2,000 ab initio structures for myoglobin as part of the example_data in the supplementary zip file. Users can test reproducibility on this dataset.
Randomization	Not applicable; no sample/organism/participant experimental groups were used.
Blinding	Not applicable; no sample/organism/participant experimental groups were used.

Reporting for specific materials, systems and methods

We require information from authors about some types of materials, experimental systems and methods used in many studies. Here, indicate whether each material, system or method listed is relevant to your study. If you are not sure if a list item applies to your research, read the appropriate section before selecting a response.

Materials & experimental systems

n/a	Involved in the study
[x]	[ ] Antibodies
[x]	[ ] Eukaryotic cell lines
[x]	[ ] Palaeontology and archaeology
[x]	[ ] Animals and other organisms
[x]	[ ] Human research participants
[x]	[ ] Clinical data
[x]	[ ] Dual use research of concern

Methods

n/a	Involved in the study
[x]	[ ] ChIP-seq
[x]	[ ] Flow cytometry
[x]	[ ] MRI-based neuroimaging
